# Baseline results from the UK SIGNIFY study: a whole-body MRI screening study in *TP53* mutation carriers and matched controls

**DOI:** 10.1007/s10689-017-9965-1

**Published:** 2017-01-16

**Authors:** Sibel Saya, Emma Killick, Sarah Thomas, Natalie Taylor, Elizabeth K. Bancroft, Jeanette Rothwell, Sarah Benafif, Alexander Dias, Christos Mikropoulos, Jenny Pope, Anthony Chamberlain, Ranga Gunapala, The SIGNIFY Study Steering Committee, Louise Izatt, Lucy Side, Lisa Walker, Susan Tomkins, Jackie Cook, Julian Barwell, Vicki Wiles, Lauren Limb, Diana Eccles, Martin O. Leach, Susan Shanley, Fiona J. Gilbert, Helen Hanson, David Gallagher, Bala Rajashanker, Richard W. Whitehouse, Dow-Mu Koh, S. Aslam Sohaib, D. Gareth Evans, Rosalind A. Eeles

**Affiliations:** 1 The Institute of Cancer Research, 15 Cotswold Road, Sutton SM2 5NG, London, UK; 2 University Hospital Southampton NHS Foundation Trust, Southampton, UK; 3 Royal Marsden NHS Foundation Trust, London, UK; 4 Central Manchester University Hospitals NHS Foundation Trust, Manchester, UK; 5 Cancer Unit, Queen Elizabeth Hospital, University Hospitals Birmingham, Birmingham, UK; 6 Guys and St Thomas’ NHS Foundation Trust, London, UK; 7 Great Ormond Street Hospital & UCL Institute for Women’s Health, London, UK; 8 Oxford University Hospitals, Oxford, UK; 9 University Hospitals Bristol NHS Foundation Trust, Bristol, UK; 10 Sheffield Children’s NHS Foundation Trust, Sheffield, UK; 11 University of Leicester, Leicester, UK; 12 University of Cambridge, Cambridge, UK; 13 Northwick Park Hospital, London, UK; 14 Peter MacCallum Cancer Centre, Melbourne, Australia; 15 St Georges Hospital, London, UK; 16 Mater Hospital, Dublin, Ireland

**Keywords:** Whole body MRI, *TP53* mutation carriers, Li Fraumeni syndrome, Controls, Screening

## Abstract

In the United Kingdom, current screening guidelines for *TP53* germline mutation carriers solely recommends annual breast MRI, despite the wide spectrum of malignancies typically seen in this group. This study sought to investigate the role of one-off non-contrast whole-body MRI (WB MRI) in the screening of asymptomatic *TP53* mutation carriers. 44 *TP53* mutation carriers and 44 population controls were recruited. Scans were read by radiologists blinded to participant carrier status. The incidence of malignancies diagnosed in *TP53* mutation carriers against general population controls was calculated. The incidences of non-malignant relevant disease and irrelevant disease were measured, as well as the number of investigations required to determine relevance of findings. In *TP53* mutation carriers, 6 of 44 (13.6, 95% CI 5.2–27.4%) participants were diagnosed with cancer during the study, all of which would be considered life threatening if untreated. Two were found to have two primary cancers. Two participants with cancer had abnormalities on the MRI which were initially thought to be benign (a pericardial cyst and a uterine fibroid) but transpired to be sarcomas. No controls were diagnosed with cancer. Fifteen carriers (34.1, 95% CI 20.5–49.9%) and seven controls (15.9, 95% CI 6.7–30.1%) underwent further investigations following the WB MRI for abnormalities that transpired to be benign (p = 0.049). The cancer detection rate in this group justifies a minimum baseline non-contrast WB MRI in germline *TP53* mutation carriers. This should be adopted into national guidelines for management of adult *TP*53 mutation carriers in addition to the current practice of contrast enhanced breast MRI imaging.

**Electronic supplementary material:**

The online version of this article (doi:10.1007/s10689-017-9965-1) contains supplementary material, which is available to authorized users.

## Introduction

Li-Fraumeni Syndrome (LFS) is a rare autosomal dominant condition which predisposes individuals to numerous cancer types. The majority of families with LFS have been found to carry mutations in the *TP53* gene [1–3]. Typical cancers in the Li-Fraumeni spectrum include soft tissue and bone sarcoma, breast, brain, adrenal cortical carcinoma and leukaemia [4], however an increased risk has also been found in many other cancer types [5, 6]. The cancers are typically young onset occurring 2 or 3 decades before the median in the general population [6], and individuals are predisposed to multiple malignancies [7]. The condition has a high penetrance, with a lifetime cancer risk of almost 100% for females and 75% for males [8] and may have a birth incidence as high as 1 in 5000 [9].

In the United Kingdom, the National Institute for Health and Clinical Excellence (NICE) recommends annual breast MRI from age 20–49 years for female *TP53* mutation carriers and to consider continuation of breast screening past age 50. Discussion of risk-reducing mastectomy is also recommended, however, no other surveillance is currently suggested in any guidelines. Screening across UK genetics centres varies and many employ an open-door policy for carriers experiencing symptoms.

Recent evidence suggests there may be a survival benefit with more intensive screening, including whole-body MRI (WB MRI) [10, 11]. Internationally, more intensive screening programmes are starting to become employed [12, 13] and, in particular, are being explored in the research setting [10, 14–16]. Several studies are employing WB MRI [14, 17], others FDG-PET/CT [15, 16], and, most commonly, combinations of several physical examination, imaging and biochemical screening modalities [10, 14, 17]. Given the lack of radiation, WB MRI provides an attractive choice to screen this cohort of individuals with an increased susceptibility to radiation-induced neoplasms [18, 19].

When considering a screening modality, the incidence of incidental findings is important in decision making whether to adopt the modality in guidelines. To our knowledge, no studies have employed a healthy control group to compare the incidence of malignancies and incidental findings to that found in the *TP53* mutation carriers.

This pilot study aimed to assess the incidence of malignancies diagnosed in asymptomatic *TP53* mutation carriers using a non-contrast WB MRI against general population controls, as well as the incidences of non-malignant relevant and irrelevant disease and the investigations required to determine relevance. It was hypothesised that more malignancies would be diagnosed in the *TP53* mutation carrier cohort than in the controls and the ratio of relevant to non-relevant findings (relevant defined as needing definitive treatment) would be higher in the *TP53* mutation carriers than controls.

## Methods

### Study subjects and data collection

From November 2012 through July 2016, *TP53* mutation carriers were recruited through genetics services from across the United Kingdom and general population controls through local advertisements. Carriers with a known low penetrance *TP53* mutation (in the view of a geneticist) or a variant of unknown significance were excluded, as well as those with a malignancy diagnosed in the previous 5 years [except non-melanomatous skin cancer or cervical carcinoma *in situ* (CIS)]. Population controls were sex and age (±5 years) matched to carriers and required to have no personal history of cancer (except non-melanomatous skin cancer or cervical CIS) and minimal family history of cancer (no first degree relative diagnosed before age 50 and only one first, second or third degree relative diagnosed at any age). Any person with current symptoms suggestive of malignancy was excluded.

An additional sub-study was included to examine the psychosocial impact of screening in this group of high-risk individuals, the results of which will be reported separately.

The research was approved by the Health Research Authority NRES Committee London—Brent (12/LO/0781).

### MRIs

MRI examinations were performed at The Royal Marsden NHS Foundation Trust or Central Manchester University Hospitals NHS Foundation Trust using a 1.5 T MRI machine (Siemens, Erlangen, Germany) from vertex to feet using conventional MR imaging sequences (T1-weighted, with or without fat suppressed T2-weighted/STIR sequences), as well as diffusion weighted images (Supplementary Table 1). Slices were 0.8 cm thick and scans were not contrast enhanced. Comparisons of imaging techniques will be reported elsewhere.

Scans were read independently by two radiologists with at least 5 years’ experience, who were blinded to the mutation status of the study participants. MRIs were divided into anatomical sections and each section scored 0 to 5 (0 normal findings, 1 definitely benign, 2 likely to be benign, 3 equivocal, 4 likely to be malignant, 5 definitely malignant). Initial recommendations for further investigations were made blinded to carrier status, however subsequent review and intervention was made with consideration of this factor. In a case of discrepancy a consensus reading was performed by the two study radiologists plus a radiologist from the other centre. Ten percent of scans, selected randomly, were cross-read at both centres for quality assurance. Lesions requiring investigation and incidental findings were discussed in a cross centre video-linked multidisciplinary team (MDT) meeting including PIs, radiologists and other study staff from both sites. All pathology was reviewed by expert pathologists in two tertiary cancer centres and at tumour-specific MDT meetings.

### Sample size and statistical analysis

A total of 88 participants were recruited to the study, with 44 carriers of *TP53* mutations matched to 44 healthy population controls. This sample size was calculated for 80% power to detect a difference in cancer detection between the mutation carrier group and the control group assuming a 20% cancer detection rate in the carrier group and 1% in the control group for a two-tailed analysis.

The efficacy of WB MRI as a screening tool was evaluated by diagnosis of early stage malignancy in asymptomatic individuals, with respect to the number of further investigations required and false positives. Incidence of malignancies with 95% confidence intervals was calculated in both carrier and control groups and any difference between groups was compared using Chi square or Fisher’s exact test as appropriate at a 5% significance level. The incidence of and proportion of the cohort with relevant and non-relevant MRI findings is reported with 95% confidence intervals. Of those who were recalled, additional investigations with non-malignant results were reported with 95% confidence intervals and any difference between groups assessed using Mann–Whitney test. All statistical tests were two-sided and analysis was performed using STATA [20].

## Results

### Patient characteristics

Forty-four *TP53* mutation carriers from 37 families and 44 matched healthy population controls were recruited (Table 1). Nine of the 27 female *TP53* carriers had had risk-reducing surgery of some kind, or mastectomies for previous breast cancer.

**Table 1:** Characteristics of the cases and controls with previous malignant tumours

	Carriers	Controls
n	44	44
Age, median (range)	38 (19–58)	38 (22–59)
Female, n (%)	27 (61%)	27 (61%)
Male, n (%)	17 (39%)	17 (39%)
Previous diagnosis of cancer, n (%)	18 (41%)	0
Breast	11*	
Sarcoma	6	
Melanoma	2	
Ovarian	1	
Wilms tumour	1	
Cervical	1	
Adrenocortical carcinoma	1	
Teratoma	1	
History of multiple cancers, n (%)	6 (13.6%)	0

*Four bilateral breast cancers; two phylloides tumours

### Cancer diagnoses and treatments

In *TP53* mutation carriers, six of 44 (13.6, 95% CI 5.2–27.4%) participants were diagnosed with cancer during the study (Table 2). Only four (9.1, 95% CI 2.5–21.7%) of these were a direct result of the study WB MRI, however two participants had two simultaneous primary tumours diagnosed. There was no diagnosis of cancer in the control group. Analysis showed no statistically significant difference in number of *TP53* mutation carriers diagnosed with malignancy compared to controls (p = 0.116), however the study was powered at a higher cancer detection rate in *TP53* mutation carriers. Table 3.

**Table 2:** WB MRI outcomes

	WB MRI outcome	Overall	Carriers	Controls
Further investigations triggered by WB MRI	Cancer detected (true positives)	4	4	0
	Eventual benign outcome (false positives)	16	9	7
	Requiring continued surveillance/Treatment (non-malignant)	3	3	0
No further investigations triggered by WB MRI	NAD (true negatives)	63	26	37
	Subsequent cancer diagnosis (false negatives)	2	2	0
Total		88	44	44

**Table 3:** Cancer diagnoses in participants

Pt	Sex	Age	Mutation	Abnormality (score) seen on WB MRI	Further investigations	Cancer	Treatment
1	F	33	c.455C > T p.Pro152Leu	Right temporal lobe cyst (4)	Dedicated brain MRI with contrast	Astrocytoma	Complete resection
2	F	51	c.659A > G p.Tyr220Cys	Left lateral abdominal wall mass—probable sarcoma (4)	US guided biopsy	Myxosarcoma	Complete resection
3	F	45	c.586C > T p.Arg196Ter	Suspicious right renal mass (4)	Abdominal CT, nephrectomy	Chromophobe renal cell carcinoma	Complete resection
				Uterine fibroids (2)	Pelvic MRI, TAH	Leiomyosarcoma	
4	F	24	c.844C > T p.Arg282Trp	Liver lesion, possible focal nodular hyperplasia or hepatic adenoma (3)	1. Dedicated renal and liver MRI with contrast. Suspected sarcomas, nephrectomy and partial hepatectomy	1. Renal EAML Liver EAML	1. Complete resection of both tumours
				Right kidney lesion, possible complex renal cyst or solid lesion (3)	2. Follow-up pelvic MRIs for EAMLs detected progressive changes in sacro-iliac joint	2. Sacro-iliac osteosarcoma	2. MAP chemotherapy completed; surgery advised but patient pursuing proton beam therapy in USA
5	F	48	c.916C > T p.Arg306Ter	Pericardial cyst (1)	Nil Non study MRI and PET revealed a 12.6 cm hilar mass with small left pleural effusion	Mediastinal liposarcoma grade 3	Resection with microscopic positive margins (0/8 lymph nodes involved) and chemotherapy
6	M	27	c.818G > A p.Arg273His	Nil	N/A	Diagnosed with B ALL (not seen on WB MRI)	Chemotherapy

*EAML* Epithelioid angiomyolipoma, *MAP* methotrexate, doxorubicin and cisplatin, *TAH* total abdominal hysterectomy

All four individuals with screen detected cancers were women who were asymptomatic and all were treated with curative intent.

The first woman had a low grade astrocytoma in the right inferior temporal gyrus and underwent a R0 resection with full post-operative recovery 2 years after surgery. Upon questioning after her diagnosis she retrospectively reported episodes of *déjà vu* but no other symptoms and had never had a cancer diagnosis previously.

The second woman was diagnosed with a myxosarcoma in the abdominal wall (Trojani grade 2). The size was 46 × 37 mm and all margins were free indicating complete surgical excision. She did not require additional chemotherapy or radiotherapy. This patient had a previous cancer history of ovarian teratoma at age 4, phylloides tumour of the breast aged 41 and DCIS breast cancer at 42 and a fibrosarcoma of the left thigh at 42.

The third woman was 45 years of age and reported irregular menstrual cycle on enrolment. She had no history of cancer and was found to have a 10.6 × 8.6 cm mass of the right kidney on WB MRI. The differential diagnosis included an oncocytoma or renal cell carcinoma. Additionally, two uterine fibroids and an ovarian cyst were detected. Following renal CT and pelvic MRI, she underwent a right nephrectomy and given her carrier status and age, simultaneous total abdominal hysterectomy, right salpingectomy (right ovary previously excised) and left salpingo-oophorectomy. A 110 mm chromophobe renal cell carcinoma (Fuhrman grade 3) confined to the kidney with no lymphovascular invasion was detected, plus an incidental benign renal angiomyolipoma measuring 15 mm. In the resected uterus, 2 fibroids were present; the larger (65 mm) was found to be a leiomyosarcoma confined to the myometrium with no evidence of vascular invasion, and the smaller (25 mm) fibroid was a benign leiomyoma. The left ovary contained a benign cyst and interestingly both fallopian tube fimbrial ends were noted to contain scattered atypical epithelial cells with severe cytological atypia, thought to be more consistent with a tubal intra-epithelial lesion in transition rather than a definite serous intra-epithelial carcinoma. Both the uterine and renal tumour resections were R0 and no further treatment was required.

The fourth participant had previously had a rhabdomyosarcoma at 6 months of age. During the study, three tumours were detected, of which the first two (in the liver and right kidney) were detected on the initial WB MRI. Subsequent dedicated liver and renal MRIs were inconclusive but were strongly suspicious of malignancy. Resection of the lesions revealed epithelioid angiomyolipomas in both organs, with suspiciously high mitotic incidence, however their malignant potential was unclear, as was the synchronicity of the tumours. Given the rarity of angiomyolipomas, tuberous sclerosis was subsequently ruled out. While on follow up for these initial tumours, a new left sacroiliac lesion was detected 19 months after the initial WB MRI. This was initially monitored then biopsied, diagnosing a high-grade chondroblastic osteosarcoma. The patient has completed methotrexate, doxorubicin and cisplatin (MAP) chemotherapy; surgery has been advised but the patient is pursuing proton beam therapy in the USA.

An additional patient had a pericardial cyst (seen on WB MRI) that was initially reported as likely benign but became symptomatic and non-study dedicated MRI and biopsy revealed a mediastinal sarcoma. The sixth was diagnosed with B-cell acute lymphocytic leukaemia 9 months after his whole-body MRI. This patient’s WB MRI was negative and he only reported some upper abdominal discomfort when questioned on enrolment in the study.

### Non-malignant findings from WB MRI

Outcomes of findings from the WB MRI are detailed in Table 2 with further details of all investigations in Supplementary Table 2. Fifteen carriers (34.1%, 95% CI 20.5–49.9%) and 7 controls (15.9%, 95% CI 6.7–30.1%) underwent further investigations after their WB MRI that did not result in a diagnosis of cancer (Table 5). There was a marginally significant difference between the groups (p = 0.049). Six carriers and one control had more than one follow-up investigation (Table 4). Of those who were recalled, *TP53* mutation carriers on average had 2.33 (95% CI 1.17–3.50) additional investigations with non-malignant results and controls 1.14 (95% CI 0.79 to 1.49). There was no significant difference between groups (p = 0.101).

**Table 4:** Multiple Investigations after WB MRI with Non-Malignant Results

	Total number additional investigations	1 investigation (n)	2 investigations (n)	3 investigations (n)	≥ 4 investigations (n)
Carriers (n = 15)	35	9^a^	1	1	4
Controls (n = 7)	8	6	1	0	0

^a^Including 2 *TP53* mutation carriers diagnosed with cancer and additional incidental findings requiring investigations

**Table 5:** Number and type of follow up investigations with Non-Malignant Results

	Overall (n = 22)	Carriers (n = 15)^a^	Controls (n = 7)
Total investigations	43	35	8
Radiation positive imaging	8	8	0
Other imaging	29^b^	21^a^	8
Biopsy/removal before definitive diagnosis	2	2^a^	0
Other investigations	4	4^a^	0

^a^Including investigations for non-malignant findings in three *TP53* mutation carriers with eventual cancer diagnoses

^b^Three scans are pending results (two *TP53* mutation carriers and one control)

There was one case in a *TP53* mutation carrier of a non-malignant incidental finding that needed intervention (triggering a rheumatology referral) and additionally three lesions in two *TP53* mutation carriers are requiring continued surveillance given their genetic status (3 of 44, 6.8%; 95% CI 1.4–18.7%).

Eight investigations with non-malignant results (four CTs, two PET-CT scans and one X-ray) were carried out in five carriers using imaging techniques that exposed the participant to radiation (Table 5). However the majority of investigations conferred no exposure: most commonly, MRI imaging was used (13 scans in carriers and two in controls, either repeat scanning to monitor growth of the lesion, addition of contrast or more detailed imaging), then ultrasound (eight scans in carriers and six in controls). Two invasive procedures that did not result in a diagnosis of cancer were undertaken in carriers in the study: a biopsy of a suspicious pelvic bone lesion and the simultaneous salpingo-oophorectomy (for likely ovarian cyst) with nephrectomy and hysterectomy in the case of the chromophobe renal cell cancer.

## Discussion

The SIGNIFY baseline WB MRI study demonstrates an overall cancer detection rate of 9.1% in prevalent WB MRI scans in *TP53* mutation carriers with no cancers identified in controls (p = 0.116). The peak annual incidence rate for malignancy in *TP53* mutation carriers is around 3% [21] therefore the prevalence in study of 9.1% suggests there is significant lead time in the cancers detected which indicates that such screening is likely to be effective in LFS. This would be further demonstrated by a low detection rate at a subsequent annual incident screens which remains to be proven. Similar findings of a high prevalent detection rate was found in the MARIBS study [22, 23] (which included *TP53* mutation carriers) with 2.7% of 632 women detected with breast cancer at prevalent screen dropping to 1.2% at the first incident round. This has translated into a significant survival benefit in women undergoing breast MRI screening [22].

The detection rate at prevalent screen could have been higher at 11.3% if further dedicated MRI had been performed as part of the study to detect the mediastinal sarcoma that had presented with a pericardial fluid collection that appeared to be a benign cyst. Additionally, the seemingly benign appearance of the uterine leiomyosarcoma also argues for a lower threshold for suspicion in this high risk cohort. Although, further investigation of these apparent benign features would lead to a higher rate of subsequent radiological investigation, this may translate into higher tumour survival rates. Complete resection of a brain astrocytoma, abdominal wall sarcoma and liver, kidney and uterine tumours may not have been possible without presymptomatic MRI detection in the current study. Sarcomas and brain malignancy in *TP53* mutation carriers have poor overall survival rates and without curative surgery patients gain little benefit from chemotherapy or radiotherapy [24–26]. Indeed the avoidance of radiotherapy through complete surgical excision may well prevent future radiation induced malignancy that appears to be very high in *TP53* mutation carriers [18]. It is likely that annual WB MRI [10, 11] will be required as sarcomas in *TP53* mutation carriers are often high grade and one patient already developed an osteosarcoma 19 months after a true negative prevalent scan.

Thus far if the missed mediastinal sarcoma and leukaemia are included, two symptomatic malignancies have developed in the 12-months post prevalent MRI. The leukaemia would not have been expected to be detected and further additional blood and other tests need to be considered in *TP53* mutation carriers as occurs in the Toronto protocol [10, 11]. In particular we do not believe that WB MRI will replace dedicated breast MRI which requires a breast coil and gadolinium injection for the highest sensitivity [23]. This indicates that both screening measures will be needed in the management of *TP*53 mutation carriers. It is not yet known how often such scans should be undertaken; at present breast MRI is performed annually from 20 to 50 years, but further studies are needed to assess if WB MRI needs to be repeated annually concurrently.

There was a high rate of identification of incidental findings at WB MRI. More than twice as many *TP53* mutation carriers required further investigation for incidental findings, resulting in a marginally significant difference between the groups (p = 0.049). Carriers who were recalled also had a higher number of repeat scans compared to controls, however this difference did not reach significance (p = 0.110). We are awaiting the psychological outcomes of these in a study related protocol, but this high rate of investigation including eight *TP53* mutation carriers versus zero controls requiring modalities involving radiation for further investigation is of concern. Nonetheless the higher rate of these findings may presage future malignancy risk as evidenced by the finding of apparent benign cystic lesions in the bone and pericardium that were found to be tumour related.

This study only considered mutation carriers with mutations previously known to be of high penetrance and it is possible that the balance of incidental findings and relevant tumour detection may not be the same in those with low penetrance mutations, such as the Brazilian founder mutation [27]. The increase in use of gene panels in families or individuals with cancer without a history of classical LFS or LFL syndrome will identify more individuals with germline *TP53* mutations where the penetrance may be lower and the role of WB MRI in such individuals is still uncertain.

LFS leads to tumours in both children and adults, however it was not possible to examine the role of screening children in the present study as the Ethics Committee passed the protocol to be undertaken in adults only. Furthermore, studies from Toronto have published the use of screening in the paediatric age group [10, 11].

The present study has some limitations. The study size was not sufficient to detect significant differences in tumour rates between cases and controls, but the trend of the data are compelling. Nonetheless the blinding of radiologists to cases and controls, the first time we are aware this has been done, is an obvious strength. In particular, the imaging of controls has demonstrated the incidental finding incidence in a small general population cohort. It is interesting that the rate of recall for further scans was higher in mutation carriers and there are animal data that suggest that mutation carriers may have dysmorphic features [28, 29]. It would be ideal to undertake an international meta-analysis of WB MRI data in *TP53* mutation carriers.

The malignancy prevalence of 13.6% in this study, detection rate on initial MRI of 9.1% and two cases of simultaneous primary cancers in two participants all argue for the adoption of at least a baseline whole body MRI scan in the screening of *TP53* mutation carriers. Given the rarity of this condition and the relative ease of delivery of MRI without contrast, this additional screen warrants further research prior to incorporation into national guidelines for management of adult *TP*53 mutation carriers in addition to the current practice of gadolinium enhanced breast MRI imaging.

## Electronic supplementary material

Below is the link to the electronic supplementary material.

Supplementary material 1 (DOCX 33 KB)
