# Supplementary material for: Baseline results from the UK SIGNIFY study: a whole-body MRI screening study in TP53 mutation carriers and matched controls
Source: Fam Cancer. 2017 Jan 16;16(3):433–40. doi: 10.1007/s10689-017-9965-1 (PMC5487773; doi:10.1007/s10689-017-9965-1)
Supplement: Supplementary file 1 — Supplementary material 1 (DOCX 33 KB) [file 10689_2017_9965_MOESM1_ESM.docx]

# Baseline Results from the UK SIGNIFY Study: A Whole-Body MRI Screening Study in *TP53* Mutation Carriers and Matched Controls

Sibel Saya MSc*, Emma Killick MD^2^*, Sarah Thomas MSc, Natalie Taylor, Elizabeth K. Bancroft PhD, Jeanette Rothwell MSc, Sarah Benafif MBBS, Alexander Dias MBBS, Christos Mikropoulos MD, Jenny Pope, Anthony Chamberlain MSc, Ranga Gunapala MSc, The SIGNIGY Study Steering Committee, Louise Izatt PhD, Lucy Side MD, Lisa Walker MS, Susan Tomkins MB ChB, Jackie Cook MD, Julian Barwell PhD, Vicki Wiles MSc, Lauren Limb MSc, Diana Eccles MD, Martin O. Leach PhD, Susan Shanley PhD, Fiona J. Gilbert FRCP, FRCR, Helen Hanson MD, David Gallagher MD, Bala Rajashanker MRCP, FRCR, Richard W. Whitehouse MD FRCR**, Dow-Mu Koh MD FRCR**, S. Aslam Sohaib MRCP FRCR**, D. Gareth Evans MD FRCP**, Rosalind A. Eeles PhD FRCP**

*Joint first authorship

**Joint last authorship

### Corresponding author:

Prof Rosalind Eeles MA, FRCR, FRCP, PhD, FMedSci

The Institute of Cancer Research and Royal Marsden NHS Foundation Trust,

15 Cotswold Road,

Sutton SM2 5NG,

United Kingdom

Tel. +44 208 722 4094

Fax +44 208 722 4110

rosalind.eeles@icr.ac.uk

Supplementary Table 1: MR Imaging Protocol

|  | **T1-weighted**  **gradient echo** | **Fat-suppressed**  **T2-weighted HASTE** | **DWIBS**  **Whole body diffusion weighted MR imaging with background signal suppression** | **T1-weighted VIBE DIXON** |
| --- | --- | --- | --- | --- |
| **Coverage** | From vertex to feet | From vertex to feet | From vertex to feet | From vertex to feet |
| **No of slice partitions** | 30 | 30 | 30 image sections per stack | 52 |
| **Technique** | Breath-hold | Breath-hold | Free-breathing | Breath-hold |
| **Orientation** | Axial | Axial | Axial | Coronal |
| **Field of View (cm)** | 38-40 | 38-40 | 38-40 | 38-40 |
| **Matrix Size** | 182 * 320 | 208 * 256 | 128 * 128 | 192 * 192 |
| **TR** | 247 | 1000 | 8600 | 6.97 |
| **TE** | 4.36 | 84 | 72 | 2.39 |
| **Flip Angle** | 70 | 180 | n/a | n/a |
| **Voxel Size (mm)** | 1.2 * 1.2 * 8 | 1.5 * 1.5 * 8 | 1.5 * 1.5 * 8 | 1 * 1 * 5 |
| **Echo-planar imaging factor** |  |  | 150 |  |
| **Parallel imaging factor** | 2 | 2 | 2 | 3 |
| **No. of signals averaged** | 1 | 1 | 4 | 1 |
| **Section thickness (mm)** | 8 | 8 | 8 | 5 |
| **Direction of motion probing gradients** | None | None | 3 scan trace | None |
| **Receiver Bandwidth** | 300 | 501 | 1954 | 450 |
| **Fat Suppression **** | None | SPAIR | STIR (T1 = 180ms) | None |
| **b-values (s/mm^2^)** | Not applicable | Not applicable | Typically 50 and 800-1000 | Not applicable |

Supplementary Table 2: Details of Incidental Findings and Follow up Investigations

| **Gender** | **Age** | **Control/Carrier** | **MRI abnormality** | **Investigations** | **Outcome** |
| --- | --- | --- | --- | --- | --- |
| M | 29 | Carrier | Maxillary sinusitis  Left leg: multiple high signals in bone  Right humerus: high T2 signal  Right leg:?deposits | Repeat WB MRI | Pending |
| M | 29 | Carrier | Left lower lobe lung lesion probably infective | Chest X-ray | Chest infection |
| M | 49 | Carrier | Thyroid nodule | Thyroid Ultrasound | Benign |
| F | 51 | Carrier | Abdominal wall lesion, suspicious of sarcoma  Liver nodule | US guided biopsy  Liver US | Myxosarcoma  Benign |
| F | 48 | Carrier | Ovarian Cysts  Pericardial Cyst | CA-125 & TVUS  None until symptomatic | Normal  Sarcoma |
| F | 48 | Carrier | 5mm lung nodule | Contrast enhanced CT chest | Lesion resolved |
| M | 41 | Carrier | Left ureteric abnormality and atrophic L kidney | Renal function (serum creatinine and estimated GFR) | Normal |
| M | 40 | Carrier | Posterior left ilium lesion | Dedicated pelvic MRI x 5  CT plus CT guided biopsy | No evidence of osteosarcoma on biopsy |
| F | 21 | Carrier | 1.8cm well circumscribed lesion segment 7 of liver  Indeterminate lesion on upper pole of right kidney 3.4 cm | Contrast MRI liver and kidney  Partial nephrectomy and partial hepatectomy | Liver and kidney EAML |
| F | 58 | Carrier | 12mm angiomyolipoma in right kidney  Possible fatty lesion in liver | Liver MRI x 2  Renal MRI x 2 + US | Continuing surveillance |
| F | 33 | Carrier | Right temporal lobe cyst | MRI brain | Astrocytoma |
| F | 34 | Carrier | Right ovarian cyst | TVUS  CA-125 x2 | Cyst resolved |
| F | 52 | Carrier | Lung lower lobe pleurally based lung lesion  Small lesion in right lobe of liver  Cystic lesion right side of vagina | Chest CT  PET-CT x2  Liver/pelvic MRI | Continuing surveillance for lung lesion  Haemorrhagic cyst of liver  Vaginal Gartner duct cyst |
| F | 53 | Carrier | 4.5 x 2.8cm cyst behind right knee | Lower leg US x 2,  MRI & CT | Benign |
| M | 43 | Carrier | Non-specific signal changes around sacroiliac joint | Pelvic MRI | Sacro-iliitis, referred to rheumatologist |
| F | 45 | Carrier | Large right renal mass  Uterine fibroids  Left ovary cyst | Abdominal CT  Pelvic MRI  Right nephrectomy, TAH and left salpingo-oophorectomy | Chromophobe renal cell carcinoma  Benign renal angiomyolipoma  Uterine leiomyosarcoma  Uterine leiomyoma  Benign left ovary cyst |
| F | 25 | Control | Right ovary cyst 3cm | TVUS | Cyst resolved |
| F | 40 | Control | Left ovary cyst 3cm | TVUS | Cyst resolved |
| M | 43 | Control | Oedema and fatty change in right gastrocnemius muscle | MRI left lower leg with contrast | Benign AVM or haemangioma |
| M | 40 | Control | Liver cyst | Liver US | Pending |
| M | 41 | Control | Liver cyst  Right renal cyst | US abdomen | Haemangioma  Simple renal cyst |
| C37  F | 39 | Control | Cyst in right SI joint  Cystic changes in breasts | MRI of right SI joint in 6 months  Breast US | Pending  Breasts- simple cysts only |
| C40  M | 51 | Control | Liver cyst haemangioma  Renal cyst | Liver and renal US | Haemangioma  Cortical cysts |

AVM = arterio-venous malformation; EAML = Epithelioid angiomyolipoma; GFR= glomerular filtration rate; SI = sacro-iliac; TAH = total abdominal hysterectomy; TV = transvaginal; US = ultrasound
